# Hepatic Involvement as the Sole Presentation of Systemic Amyloid Light Chain (AL) Amyloidosis: A Diagnostic Challenge

**DOI:** 10.7759/cureus.47310

**Published:** 2023-10-19

**Authors:** Nadeer Kottavadakkeel, Arun Rajaram

**Affiliations:** 1 Gastroenterology, Pilgrim Hospital Boston, United Lincolnshire Hospital NHS Trust, Boston, GBR; 2 Gastroenterology, Aster Medcity, Kochi, IND; 3 Otolaryngology, Pilgrim Hospital Boston, United Lincolnshire Hospital NHS Trust, Boston, GBR

**Keywords:** apple-green birefringence, congo red stain, hepatic amyloidosis, infiltrative liver disease, al amyloidosis, hepatomegaly

## Abstract

Primary systemic amyloidosis is generally a systemic condition and only a few of systemic amyloidosis cases manifest with signs of single-organ involvement. The occurrence of symptoms and signs of hepatic involvement alone in primary systemic amyloidosis is rare, presenting a diagnostic challenge. In this case, a 55-year-old lady presented with nonspecific symptoms such as weight loss and loss of appetite. She was found to have mildly deranged liver function tests with a cholestatic pattern, and there were no apparent risk factors for liver disease. Clinical features of involvement in other organs were notably absent. After ruling out common causes of cholestatic liver disease, we considered the possibility of infiltrative liver disease and arranged for a liver biopsy, which revealed the diagnosis of amyloidosis.

In summary, while hepatic deposition is a relatively common consequence of systemic amyloidosis, it is exceptionally rare for a patient to present with clinical features of liver involvement alone. This rarity presents a significant diagnostic challenge. Given the infrequency of this presentation, a diagnosis of amyloidosis should be considered only after diligently excluding other more common causes of hepatomegaly, whether associated with abnormal liver function tests or not.

## Introduction

Rudolph Virchow, in 1854, introduced and popularized the term *amyloid* to denote a macroscopic tissue abnormality that exhibited a positive iodine staining reaction. Amyloidosis occurs due to the extracellular deposition of toxic fibrillar amyloid protein. The subsequent enlargement and impaired function of affected organs have piqued the interest of both pathologists and clinicians. It can be either hereditary or acquired in nature. Amyloidosis typically presents as a multisystemic disease; however, isolated organ involvement can also rarely occur. The most prevalent deposit types are AL, where A represents amyloid and L signifies light chain, amyloid serum A protein (AA), amyloid transport protein transthyretin (ATTR), and dialysis-related amyloidosis involving beta-2 microglobulin [1,2]. AL amyloidosis is considered a plasma cell disorder caused by a generally small and slowly proliferating clone of plasma cells in the bone marrow that produces nonfunctional immunoglobulins, which misfold to form β-pleated amyloid fibrils [3]. Hepatic involvement as the only manifestation of primary systemic amyloidosis is a rare occurrence. It is characterized by liver disease that leads to an enlarged liver (hepatomegaly) and abnormal liver function, resulting in a cholestatic blood profile. This condition is often accompanied by constitutional symptoms such as weight loss. Our case report is about a patient who presented with systemic AL amyloidosis with liver involvement as the sole presentation, posing a diagnostic challenge.

## Case presentation

A 55-year-old woman, known for biopsy-proven lymphocytic thyroiditis, hypothyroidism, and systemic hypertension (HTN), was referred to the gastroenterology outpatient clinic due to presenting symptoms of weight loss (approximately 6 kg over six months), abdominal fullness, and reduced appetite. Notably, she did not exhibit jaundice, hematemesis, melena, abdominal distension, or changes in bowel habits. She had no history of alcohol consumption or chronic conditions such as tuberculosis, rheumatoid arthritis, or chronic lymphocytic leukemia (CLL).

Initial assessments were conducted elsewhere and included blood tests, imaging studies, and endoscopy. The findings were as follows: abnormal liver function tests, with elevated bilirubin at 25 umol/L (reference range: <20.5 umol/L), and increased alkaline phosphatase (ALP) at 270 IU/L (reference range: 35-104 IU/L). Imaging showed evidence of hepatomegaly with fatty liver disease without any other significant abnormalities on ultrasonography. Endoscopy revealed antral erosions with negative results on rapid urease tests and nonspecific histological findings in the upper gastrointestinal tract.

The patient's observations remained stable, and the general examination did not reveal any noteworthy findings. During the abdominal examination, significant, non-tender, and firm to hard hepatomegaly was noted. The hepatomegaly extended about four fingerbreadths below the right costal margin, and the liver span measured 17 cm. There was no indication of splenomegaly or ascites. However, a urine routine was conducted using the protein error of indicator principle on a spot urine sample, revealing no detectable protein or albumin, consistent with the provided reference interval of nil.

A follow-up blood test conducted at the time of presentation showed normal urea and electrolyte levels (Table 1). Total and direct bilirubin levels were 17 umol/L and 7.86 umol/L, respectively. However, ALP levels had further increased to 332 IU/L, while transaminase levels remained within the normal range. Additionally, the total protein was measured at 7.34 g/dL, albumin at 4.63 g/dL and the international normalized ratio (INR) was 1.03. Laboratory findings also indicated thrombocytosis, leucocytosis with mild lymphocytosis, and a mild hypochromic microcytic blood picture.

**Table 1:** Routine blood test results

Parameter	Result	Reference Range
Serum urea	25.2 mg/dL	10-40 mg/dL
Creatinine	0.83 mg/dL	0.6-1.1 mg/dL
Total bilirubin	17 umol/L	<20.5 umol/L
Direct bilirubin	7.86 umol/L	<3.42 umol/L
Alkaline phosphatase	332 IU/L	35-104 IU/L
Alanine transaminase (ALT)	21.5 IU/L	<34 IU/L
Aspartate transaminase (AST)	28.4 IU/L	<31 IU/L
Total protein	7.34 g/dL	6.4-8 g/dL
Albumin (A)	4.63 g/dL	3.6-5.1 g/dL
Globulin (G)	2.71 g/dL	2.3-3.5 g/dL
A/G ratio	1.71	1.1-2.2
International normalized ratio (INR)	1.03	0.8-1.2
Platelet count	631 x 10^9^/L	150-410 x 10^9^/L
Leukocyte count	12.37 x 10^9^/L	4-10 x 10^9^/L
Lymphocyte count	6.28 x 10^9^/L	1-3 x 10^9^/L
Hemoglobin	110 g/L	120-150 g/L
Mean corpuscular volume (MCV)	81.6 fL	83-101 fL
Mean corpuscular hemoglobin (MCH)	24.4 pg	25-33 pg

The viral hepatitis screen for hepatitis C virus (HCV), hepatitis B virus surface antigen (HBsAg), and human immunodeficiency virus (HIV) test results were non-reactive. The autoimmune liver disease screen, ferritin level, and alpha-1 antitrypsin (AAT) levels all fell within the normal range. The QuantiFERON-TB Gold test yielded a negative result. Additionally, alpha-fetoprotein (AFP) levels were within the normal range.

**Figure 1:**
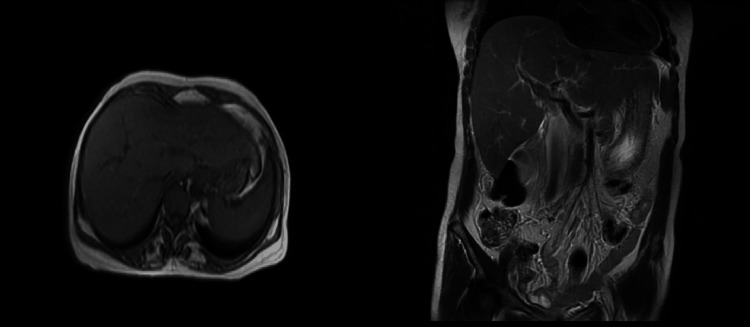
Abdominal MRI images in the T2-weighted coronal and sagittal sections indicating hepatomegaly with fatty liver

**Figure 2:**
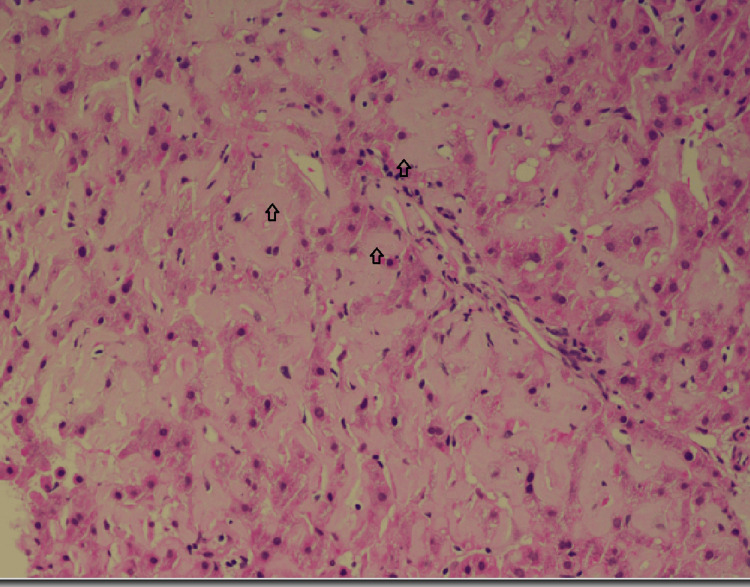
Liver biopsy with H&E staining (magnification, 20x) The arrows show glassy homogeneous eosinophilic materials. H&E, hematoxylin and eosin

**Figure 3:**
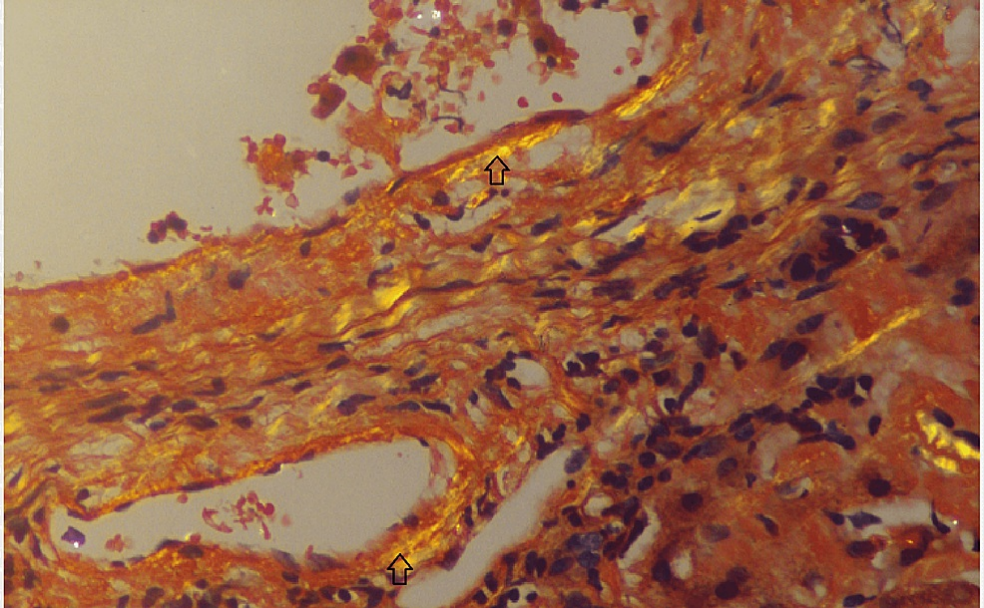
Liver biopsy with Congo red staining (magnification, 20x) The arrows show areas of apple-green birefringence on Congo red staining, suggestive of amyloidosis in the liver.

**Figure 4:**
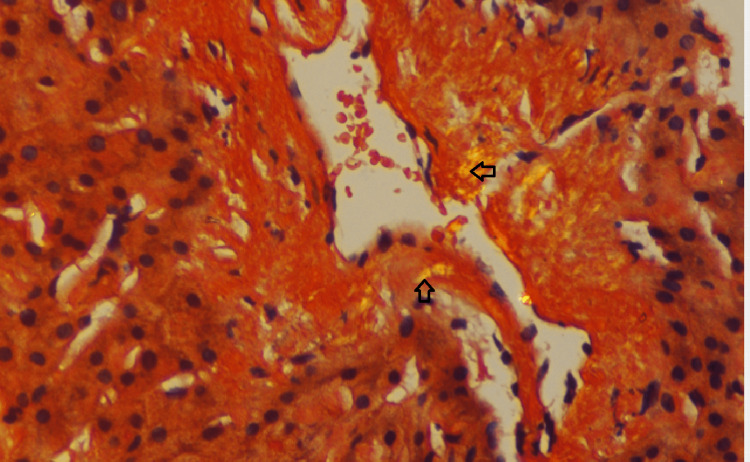
Liver biopsy with Congo red staining (magnification, 40x) The arrows show areas of apple-green birefringence on Congo red staining on the vessel wall, suggestive of amyloidosis in the liver.

An abdominal magnetic resonance imaging (MRI) scan indicated hepatomegaly with a liver span of 17 cm without signs of chronic parenchymal liver disease, biliary obstruction, or portal vein thrombosis (Figure 1). Given the hepatomegaly coupled with a cholestatic presentation and the absence of evidence suggesting biliary obstruction, a liver biopsy was conducted to explore the possibility of infiltrative liver disease. The biopsy demonstrated significant atrophy of liver cell plates along with the deposition of acellular, glassy, homogeneous eosinophilic material in the sinusoids, portal areas, and vessel walls (Figure 2). This material did not exhibit positive staining with periodic acid-Schiff (PAS) stain; however, it displayed an apple-green birefringence under the Congo red stain (Figures 3, 4). These findings concurred with a diagnosis of hepatic amyloidosis.

The hematological investigations, including serum protein electrophoresis, effectively ruled out multiple myeloma and monoclonal gammopathy. In the serum immunofixation electrophoresis (IFE) analysis, there was no evidence of monoclonal gammopathy observed, as indicated by the absence of IgG band, IgM band, IgA band, kappa band, lambda band, or M-spike. The bone marrow aspirate showed trilineage hematopoiesis with normocellular normoblastic marrow (plasma cells were 1% of the total count). Myeloma-defining events, as outlined by the International Myeloma Foundation, were absent in our case. There were only 1% clonal plasma cells observed in the bone marrow examination, and the serum involved/uninvolved free light chain (FLC) ratio was 8.4, which is significantly below the required 100 or greater for diagnosis. Additionally, there was no reported bone pain or suspicion of bony lesions in our case.

The serum FLC assay displayed a kappa value of 29.4 mg/L (reference range: 3.3-19.4) and a lambda value of 247 mg/L (reference range: 5.7-26.3), resulting in a kappa/lambda ratio of 0.12 (reference range: 0.26-1.65). This ratio suggested AL type amyloidosis.

The bone marrow aspirate revealed trilineage hematopoiesis with a normocellular and normoblastic marrow, with plasma cells accounting for only 1% of the total count. No amyloid deposits were observed. Additionally, a chromosomal analysis was performed on the bone marrow aspirate to rule out mutations specific to myeloproliferative neoplasms or myelodysplastic syndromes, and it indicated a normal karyotype. A fluorescence in situ hybridisation (FISH) test was not conducted due to the low percentage (1%) of plasma cells noted in the marrow. Furthermore, a DNA test performed on the blood to identify mutations indicative of myeloproliferative neoplasms returned negative results, confirming the absence of such mutations.

The echocardiogram only revealed concentric left ventricular hypertrophy, likely due to the patient's existing systemic HTN. No features suggestive of cardiac amyloidosis on the echo, such as interventricular septal thickening or restrictive cardiomyopathy, were observed. The B-type natriuretic peptide (BNP) level was measured at 78.8 pg/mL (reference range: 100 pg/mL), and the troponin I level was 3.74 ng/L (reference range: <9).

With an organ-specific biopsy report already showing amyloidosis and a serum light chain assay indicating elevated lambda levels, the hematologist confirmed a diagnosis of systemic AL amyloidosis, presenting as infiltrative liver disease classified as modified Mayo Stage 1 disease. Subsequently, the patient underwent cyclophosphamide, bortezomib, and dexamethasone (CyBorD) chemotherapy (daratumumab was costly, and the patient decided against seeking treatment at a government hospital for potential cost savings, as they were uncertain about the medicine's availability there at that time). Later, she underwent autologous stem cell transplantation (ASCT), and has been progressing well. It has been one and a half years since the diagnosis of the disease.

## Discussion

Amyloidosis is typically a systemic condition; only 10%-20% of cases of primary amyloidosis present with features of single-organ involvement. Signs and symptoms of hepatic involvement alone in amyloidosis are rare and often require invasive diagnostic procedures like a liver biopsy. The majority of initial symptoms are nonspecific, delaying diagnosis and resulting in progressive organ dysfunction and ultimately irreversible failure [4]. Hepatic dysfunction tends to manifest subclinically, featuring hepatomegaly, mild jaundice, and, in rare cases, severe cholestasis. Notably, hepatic amyloidosis carries the risk of spontaneous hepatic rupture and significant hemorrhage. Therefore, the performance of needle biopsies should be approached with utmost care [5].

A study involving 98 patients with primary systemic amyloidosis and confirmed hepatic involvement through biopsy demonstrated that several markers could point towards a diagnosis of hepatic amyloidosis. These markers include hepatomegaly, unexplained elevation in serum alkaline phosphatase levels, unintended weight loss, proteinuria, increased serum or urinary monoclonal protein, and evidence of hyposplenism on a blood smear (indicated by Howell-Jolly bodies) [6].

 A total of 7%-8% of patients with amyloidosis have an underlying autoimmune disease. Monoclonal paraproteinemia can co-occur with non-AL subtypes of systemic amyloidosis or in patients without amyloidosis at all (i.e., patients with monoclonal gammopathy of undetermined significance, or MGUS). Multiple myeloma and monoclonal gammopathy were effectively ruled out in our patient as mentioned in the case presentation.

To check for systemic amyloidosis, the first step is to look for a certain type of abnormal protein in the blood and urine. This is done through electrophoresis and immunofixation tests on both serum and urine, examining serum kappa and lambda FLC levels, and measuring 24-hour urine protein [7,8]. If these tests confirm the presence of an abnormal protein or an unusual FLC ratio, it strongly suggests AL amyloidosis. A prompt tissue biopsy is crucial to confirm the diagnosis [9]. Noninvasive methods such as organ-specific serum biomarkers and imaging studies can be used to obtain valuable information about the organs involved [10].

Congo red stain is the gold standard tissue staining technique to diagnose amyloidosis. The amyloid fibrils appear as green birefringent areas under a polarized light microscope [11]. The site of biopsy can be an end organ with suspected fibril deposition or a surrogate tissue (the abdominal fat, bone marrow, or salivary gland) as amyloid often deposits in these areas [12]. The choice of the biopsy site frequently depends on the institutional experience and clinician preference. The combination of abdominal fat aspiration and bone marrow biopsy allows for both the evaluation of the underlying plasma cell dyscrasia and increased sensitivity of diagnosis [9]. Both procedures are safe, fast, and relatively simple and have a sensitivity of up to 85% [13]. In the context of a negative surrogate site biopsy but persistent clinical suspicion of AL amyloidosis, target organ biopsy should be pursued [14]. If amyloid is identified on biopsy, determination of the type of amyloid should be performed for complete diagnosis. Several methods can be used for amyloid typing including immunohistochemistry, electron microscopy, and laser microdissection with mass spectrometry-based proteomic analysis [9,12]. The latter is considered the gold standard as it can precisely type all known subtypes of amyloid, including the very rare ones; however, it is not widely available [15].

In healthy patients who opt for targeted treatment, a combination of four antiplasma cell drugs is employed to treat newly diagnosed AL amyloidosis. This includes daratumumab, cyclophosphamide, bortezomib, and dexamethasone (referred to as CyBorD) [16]. The rationale behind this approach is supported by the recent phase III ANDROMEDA study [17].

Currently, for eligible patients considering ASCT, the recommendation is to start with daratumumab-CyBorD induction therapy for two to four cycles [18], particularly for patients with a plasma cell count of 10% or higher. After this, the response is evaluated. If the patient achieves a hematologic very good partial response (VGPR) or better [18], we skip ASCT and its associated treatment risks, opting to complete daratumumab-CyBorD induction and then transition to daratumumab maintenance for a total of two years. In cases of a partial response, two additional cycles of treatment are administered and ASCT is planned for if a VGPR is not attained [19]. Lastly, if there is progression of the disease, it is decided to proceed to second-line therapy. Once a hematologic response is achieved, it is important to discuss the option of a liver transplant if there is no organ recovery or if there is progressive liver failure.

Our case was challenging as the patient presented with nonspecific symptoms such as weight loss and loss of appetite. She was found to have mildly deranged liver function tests with a cholestatic pattern, and there were no apparent risk factors for liver disease. Clinical features of involvement of other organs were notably absent. After ruling out the common causes of cholestatic liver disease, we considered the possibility of infiltrative liver disease and arranged for a liver biopsy, which revealed the diagnosis of amyloidosis. In the absence of systemic features (such as renal, gastrointestinal, or neuronal involvement) and a chronic infective or inflammatory state, this diagnosis could have been entirely missed if the liver biopsy had not been performed. The diagnosis of systemic amyloidosis is typically more straightforward with less invasive tests, such as subcutaneous fat or rectal biopsy.

## Conclusions

In summary, while hepatic deposition is a relatively common consequence of systemic amyloidosis, it is exceptionally rare for a patient to present with clinical features of liver involvement alone. This rarity presents a significant diagnostic challenge. Given the infrequency of this presentation, a diagnosis of amyloidosis should be considered only after diligently excluding other more common causes of hepatomegaly, whether associated with abnormal liver function tests or not.
